# The external scent efferent system of selected European true bugs (Heteroptera): a biomimetic inspiration for passive, unidirectional fluid transport

**DOI:** 10.1098/rsif.2017.0975

**Published:** 2018-03-28

**Authors:** Florian Hischen, Gerda Buchberger, Cristina Plamadeala, Oskar Armbruster, Ernst Heiss, Kai Winands, Martin Schwarz, Bert Jüttler, Johannes Heitz, Werner Baumgartner

**Affiliations:** 1 Institute for Biomedical Mechatronics, Johannes Kepler University Linz, Altenberger Straße 69, 4070 Linz, Austria; 2 Institute for Applied Physics, Johannes Kepler University Linz, Altenberger Straße 69, 4070 Linz, Austria; 3 Institute for Applied Geometry, Johannes Kepler University Linz, Altenberger Straße 69, 4070 Linz, Austria; 4 Tiroler Landesmuseum, Josef-Schraffl-Straße 2a, 6020 Innsbruck, Austria; 5 Fraunhofer Institute for Production Technology IPT, Steinbachstraße 17, 52047 Aachen, Germany; 6 Oberösterreichisches Landesmuseum, Johann-Wilhelm-Klein-Straße 73, 4040 Linz, Austria

**Keywords:** biomimetics, true bugs, liquid–surface interaction, microfluidics, passive fluid transport, directional fluid transport

## Abstract

In this work, we present structured capillaries that were inspired by the microstructures of the external scent efferent system as found in different European true bug species (Pentatomidae and Cydnidae). These make use of small, orientated structures in order to facilitate fluid movement towards desired areas where defensive substances are evaporated. Gland channels and microstructures were investigated by means of scanning electron microscopy and abstracted into three-dimensional models. We used these models to create scent channel replicas from different technical substrates (steel and polymers) by means of laser ablation, laser structuring and casting. Video analysis of conducted fluid-flow experiments showed that bug-inspired, artificial scent fluid channels can indeed transport different fluids (water solutions and oils/lubricants) passively in one direction (velocities of up to 1 mm s^−1^), while halting the fluid movement in the opposite direction. At the end of this contribution, we present a physical theory that explains the observed fluid transport and sets the rules for performance optimization in future work.

## 1. Introduction

The interaction of liquids and surfaces has always been of great interest for various fields of research and application. Recently, for example, the field of microfluidics with emerging applications like lab-on-a-chip (e.g. [1]) has attracted increasing interest not only for better understanding of basic physical principles, but also for solutions that allow the guidance of fluids in a controlled and predictable fashion [2].

History has shown that nature has brought forth solutions for most complex problems—some already understood and applied, others discovered but not yet understood and the majority probably undiscovered—therefore, giving the field of biomimetics growing relevance in the past few decades. Prominent findings, e.g. the discovery of the lotus effect [3,4], fog harvesting, inspired by beetles or the webs of spiders [5,6], show only a glimpse of liquid–surface interaction that can be found in nature and that in some cases leads to revolutionary applications. Hence, having a closer look at nature's various ‘liquid-specialists’ will provide even more insight towards this end—from expanding the understanding of basic principles to new fields of applications.

In nature, surface–liquid interaction can be found everywhere. Not only in aquatic environments is the phenomenon constantly present; all terrestrial life is equally concerned. Body surfaces interact with fluids all the time and everywhere. In many cases, this interaction concerns water, for example, from rain or dew, or water-based fluids and solutions (e.g. sweat) on the skin or shell of an animal. There are many examples that go beyond simple passive interaction. Nature has come up with a variety of systems that allow for influencing of body-surface fluids in very specific ways: lizards harvesting moisture and directing fluid passively to their mouths [7,8] and female fleas being able to direct fluids passively within their spermatheca [9], or camouflage based on getting wet or staying dry, as observed on the neotropical flat bug *Dysodius magnus* [10,11], are only a few examples for this. Especially when we have a closer look into the class of insects, the most common interaction is the repellence of water on the surface. Owing to a cuticle that mostly consists of waxes and non-polar substances [12,13], most insects are bestowed with a hydrophobic surface and, therefore, water will form droplets, which then eventually roll off. Additionally, it has been shown that the hygroscopic response of insect surfaces can be majorly affected by microstructures such as hairs and setae [14,15]. These mechanisms provide insects with an effective protection against rain and dew droplets that would otherwise render them immobile, or even block their tracheas.

In addition to described water interactions, there are oil/fat-based fluid interactions on plants and also animals. Here we talk in most cases about oily substances that are produced from the organisms themselves. Examples for such fluids can be found on nearly every higher life form, for instance fat secretion on human skin/hair [16], prevention of wetting, e.g. the uropygial glands of water birds [17], or, as in the case of many insects, defensive secretions with the purpose of fending off and/or distracting predators (e.g. [18,19]).

Especially, the order of Hemiptera and the sub-order of Heteroptera are well known for showing a wide selection of species that produce oily defensive fluids in order to defend themselves. Probably one of the best known examples for this is the common green stink bug, *Palomena prasina* (Linnaeus, 1761), which produces sticky and intensively smelly defensive secretions from metathoracic scent glands located ventrally between the meso- and meta-legs (e.g. [20,21]). This, as well as most bug defensive fluids, for the main part consist of *n*-alkanes and *n*-alkenes, as well as aldehydes and alkenes (e.g. [18,19,22]). Such fluids often are guided through channel systems or capillaries to reach areas of the cuticle where evaporation can take place most efficiently. Such evaporative surfaces (as part of the metathoracic scent gland apparatus; cf. [23]) are usually characterized by microscale protrusions, wrinkles or microstructures that increase surface and allow for more effective evaporation of the active repellent substances in the defensive fluid [23,24]. A well-described example for these are the evaporation surfaces of *Graphosoma lineatum* (Linnaeus, 1761) [25].

It has already been shown that one of the interesting elements of the defensive secretion system in true bugs is the channel guiding the fluid to the above-mentioned evaporation surfaces, the peritreme. Plamadeala *et al*. [26] showed that a microstructured fluid channel of the neotropical flat bug species *Dysodius* can be used as an biomimetic inspiration towards the design of technical surfaces that transport oily fluids unidirectionally. However, in the case of *Dysodius*, the transport system functions in the form of a closed capillary, because its wings cover the channel structure on the bug itself.

**Figure 1.**
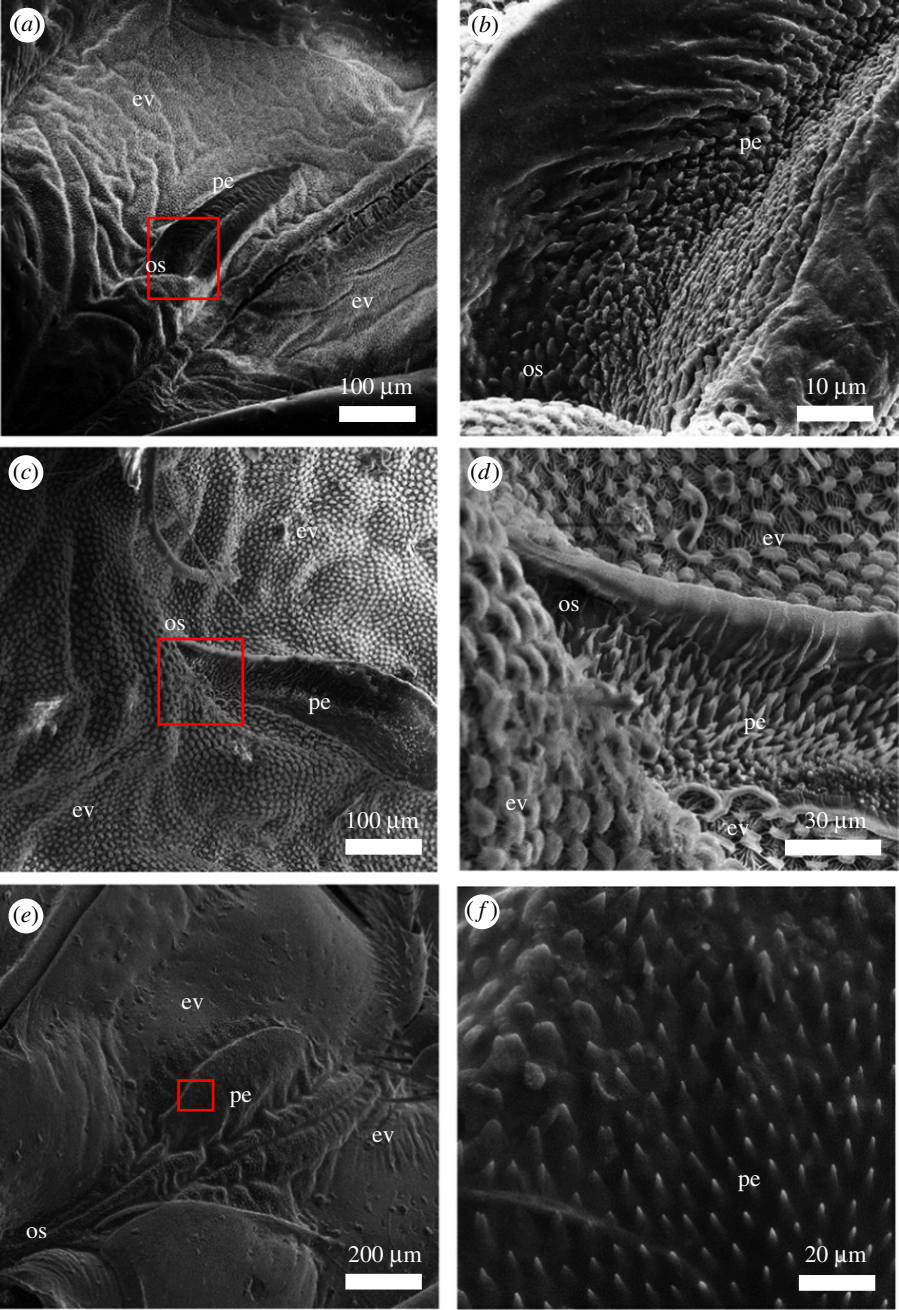
SEM overview of investigated species' ESES. Depicted parts of this system are the ostiole (os), the peritreme (pe) and the evaporatorium (ev). (*a*) *Palomena prasina*. (*b*) Magnified box from (*a*), showing a detail of the orientated microstructure of the peritreme close to the ostiole. (*c*) *Rhaphigatser nebulosa*. (*d*) Magnified box from (*c*), again depicting the microstructure of the peritreme close to the ostiole. (*e*) *Tritomegas bicolor*. (*f*) Magnified box from (*e*), showing a detail of the orientated microstructure of the peritreme. In this case, the structure is located away farther from the ostiole than in (*a*) and (*c*), closer to the edge of the evaporatorium.

By contrast, the focus of this contribution was to find such transport systems in an open-channelled form. Inspired by the findings for *Dysodius* and the comprehensive work of Kment & Davidová-Vilímová [23] about external scent efferent systems (ESES) of pentatomids, we searched for species that exhibit modified structures within their defence gland systems that might contribute towards directed liquid transport. Towards this end, we screened through a variety of European true bugs with regard to their microscopic features of the ESES. Preselection left us with three promising species from the families of Pentatomidae and Cydnidae. *Palomena prasina*, *Rhaphigaster nebulosa* (Poda, 1761) and *Tritomegas bicolor* (Linnaeus, 1758) have in common that they all show microstructured areas of either (i) the direct surroundings of the scent glands' orifices or (ii) the pathway connecting orifice and evaporative areas. An overview of the species and the respective microstructures is given in figure 1. Connatural to all found structures is the always observable orientation of the tips away from the orifice, as well as the similarity in the size and aspect ratio. This gave reason to the idea that the observed structures contribute to the directionality of secreted defensive fluids, as has been previously shown for arrays of asymmetric structure patterns in capillaries (see above).

In this work, we extracted the particular structural features of the mentioned true bug species and converted them into abstracted models. Arrays of this structure were then superimposed into channels on different artificial materials (steel and polymers) and tested with respect to their influence on liquid–surface interaction of oily and watery fluids. The observed fluid movement was video-captured and analysed with specialized algorithms with regard to directionality and speed.

## 2. Experimental

### 2.1. Abstraction of the fluid channel and transfer to a technical surface

**Figure 2.**
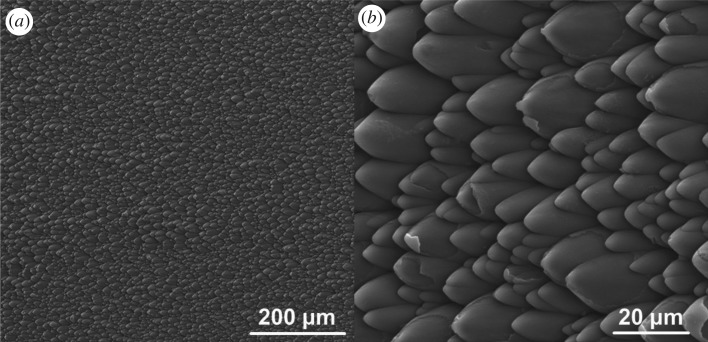
Laser-structured PI foils (angle of incidence = 45°). (*a*) Overview and (*b*) magnification of (*a*).

Scanning electron microscopy (SEM) images from the ESES of *P. prasina*, *R. nebulosa* and *T. bicolor* were obtained with a Philips 525 SEM (Philips, Germany). Two dead and air-dried specimens of each species were dried in an ascending ethanol series (50–100%) and afterwards sputter-coated with gold (former Polaron Unlimited, UK). Coating took place at 10 mA and 1 kV for 180 s in order to achieve a coat thickness of 20 nm. Images of the respective scent gland of each species and the adjusted channel and evaporative structures were taken (figure 1), and the spikey structures found within the peritremes were measured by means of length, width and slope inclination, using the freeware Fiji (a plugin packed version of ImageJ [27], website: https://imagej.net/Fiji; used version: v. 1.51p). Measurements of the individual microstructures of all three investigated species revealed that the structures show average lengths between 6 and 12 µm, average widths at their bases between 2 and 7 µm, and average heights of around 2–5 µm. The centre to centre distance between individual structures is approximately 10 µm (*n* = 15–25 measurements for each species and parameter). A better way, though, to uniformly describe these structures is the usage of aspect ratios. We concluded average aspect ratios of 2.3(±0.26) : 1 (length : width) and approximately 4.3(±0.15) : 1 (length : height). These ratios were used to abstract the microstructure into a first three-dimensional modelled array with the aim to reproduce an artificial channel as close as possible to the original scale (see ‘Laser-structured polyimide foils’). Additionally, we used the same aspect ratios afterwards to create an upscaled three-dimensional model, suitable for laser ablation. For the actual model used in the laser process, individual structures were composed into a symmetric array and placed at the bottom of a capillary channel with the dimensions of 5 × 1 cm^2^ and a depth of 100 µm (figure 2*b*). The abstracted model was laser-engraved into a hardened piece of working steel. A second steel plate was engraved with the same model, but with inverted *z*-dimensions, in order to achieve a negative version of the structure that could be used as a mould for producing polymer replicas.

### 2.2. Laser-structured polyimide foils

A first real-scale attempt to transfer the microstructures found in the ESES onto technical surfaces was done by laser treatment of polyimide (PI) foils. Used PI foils had a surface area of 3.5 × 3.5 cm^2^, density 1.47 g cm^−3^ and Tg > 500°C, and were supplied by UBE Industries, Ltd. Prior to mounting the substrate on the holder, the foils were cleaned with ethanol in an ultrasonic bath for 30 min. A KrF excimer laser (LPX 300, former Lambda Physik, now Coherent Deutschland GmbH, Germany, wavelength of 248 nm, pulse duration of 20 ns, at a repetition rate of 1 Hz) was used for PI surface structuring. To obtain a larger area covered with tilted conical microstructures, the sample holder was tilted at 45° and horizontally moved at a speed of about 16.4 µm s^−1^. For one sample around 2100–2200 pulses were used, with 255 mJ of energy. After the laser processing, PI foils were cleaned with ethanol in an ultrasonic bath for 30 min. An example overview of the surface properties of laser-structured PI foils is shown in figure 2. The structured area had a size of 2.5 × 1 cm^2^.

### 2.3. Steel replicas of the scent gland channel

First, demonstrator plates with the bug-inspired structures were manufactured using the process of laser ablation. Therefore, an ultra-short pulsed laser (SuperRapid, previously LUMERA LASER GmbH, now Coherent Deutschland GmbH, Germany), integrated in a 5-axis precision machine (KERN Microtechnik GmbH, Germany), was used. The laser source emitted a pulsed laser radiation with a wavelength of 532 nm and constant pulse duration of 9 ps. A laser scanner, intelliScan10, and a dynamic beam expander, VarioScan20i, realized the beam guidance (both SCANLAB GmbH, Germany). The laser beam was focused on the surface of the demonstrator plates using a telecentric f-theta lens (Qioptiq Photonics GmbH & Co. KG, Germany) with a focal length of 100 mm. The base material of the plates was heat-treated steel X37CrMoV5-1 with a hardness of 50 HRC. The laser structuring of the steel material was finally performed with a pulse energy of 12.5 µJ achieved by selecting an average laser power of 5 W and a pulse repetition rate of 400 kHz. The active feed rate of the laser beam was 500 mm s^−1^. The programming and calculation of the laser paths was done with the special CAM software ‘CALM’, developed at the Fraunhofer IPT. All the programs for laser surface structuring included a contour-following strategy with a defined path overlap of 10 µm. The effective ablation depth per single laser pulse reached was 0.75 µm at the positive bug structure and 1.25 µm at the negative bug structure.

### 2.4. Poly(methyl methacrylate) replica of the scent gland channel

The negative-patterned steel plate was used as a mould to create a polymer replica of the channel structure from poly(methyl methacrylate) (PMMA) by means of hot-embossing. PMMA pellets (Lucite Diakon, Lucite International, The Netherlands) were placed into a steel bowl, distributed evenly and the mould plate (laser-ablated steel negative) was placed on top. The structured side thereby faced the PMMA pellets. Both the steel bowl and the negative steel mould had been thoroughly cleaned with ethanol and lint-free cloths. Onto the mould, a steel weight (13 kg) was placed to supply pressure for the hot-embossing process. This hot-embossing composition was incubated in a vacuum drying chamber (Binder VD115, Binder, Germany). The chamber was heated to a final temperature of 210°C and evacuated to a pressure of 250 mbar, using an Edwards Stage 5 E2M5 dual stage mechanical vacuum pump (Edwards Limited, Atlas Copco Group, Sweden). The vacuum served the purpose of evacuating gas from the PMMA and, therefore, achieved a bubble-free result and prevented oxidation. After 2 h of incubation, heating was switched off and the chamber was opened for cooling. The PMMA sample was left to slowly cool down for 45 min in the chamber. Before the embossing mould was removed, the embossed PMMA was cooled for an additional 10 min outside the chamber at room temperature. After this, the PMMA replica of the scent gland channel was removed from the steel bowl and ready to be tested.

### 2.5. Poly(dimethylsiloxane) replica of the scent gland channel

The same steel mould as described above was also used to create a polymer replica from poly(dimethylsiloxane) (PDMS; Sylgard 184 Silicone Elastomer Kit, Dow Corning, USA). The mould was placed into a glass Petri dish, with the structured side facing upwards. A mixture from base and hardener was prepared according to the data sheet and degassed in a vacuum bell jar. The set-up was carefully levelled in order to avoid thickness inhomogeneity and afterwards poured with the PDMS mixture. A needle was used to get rid of bubbles. The poured PDMS was then set to cure for 3 h at 50°C and then left for 24 h at room temperature, before the replica was cut out and retracted from the mould.

### 2.6. Quality survey of the replicas

**Figure 3.**
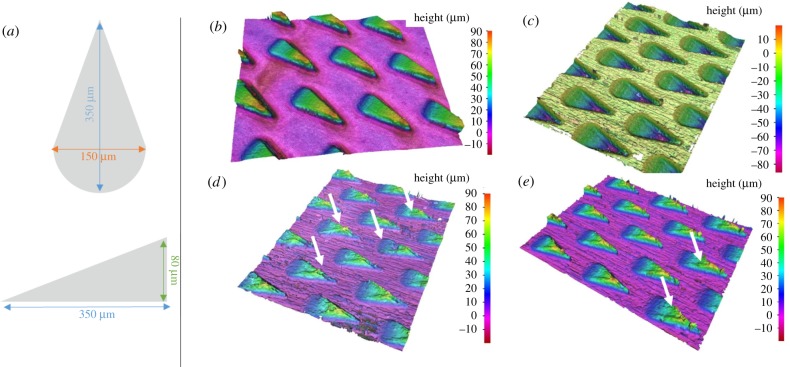
Model and outcome of laser ablation-based prototypes. (*a*) Dimensions for an individual microstructure. The tip-to-base distance between two structures within one row was set to 350 µm, while the centre-to-centre distance for two neighbouring rows was set to 200 µm. (*b–e*) Results of the optical quality survey done by Alicona. (*b*) Laser-structured steel prototype with positive microstructure. (*c*) Laser-structured steel prototype with negative microstructure. This prototype was used as a mould to cast (*d*) and (*e*). (*d*) Cast PMMA prototype. Arrows indicate example flawed areas of the structure. (*e*) Cast PDMS prototype. Arrows indicate example flawed areas of the structure.

Laser-structured PI foils were surveyed by means of SEM (same device as stated above), while all upscaled replicas—especially their surface topography—were analysed with a three-dimensional microscope: Alicona Infinite Focus G4 (Alicona Imaging GmbH, Austria). This microscope enables detailed surface measurements with resolutions up to 30 µm in the *x*–*y*-direction and 10 nm in the *z*-direction, using white light interferometry and the patented Alicona Focus Variation Technology. Figure 3 summarizes the design aims for all laser ablation-based prototypes, as well as the outcome of the quality survey.

All structures in images obtained by Alicona or SEM were measured using the freeware ImageJ.

### 2.7. Testing and analysing the fluid behaviour

All produced replicas of the bug-inspired channel (PI foils, steel, PMMA, PDMS) were tested with different fluids in order to see fluid movement or fluid–surface interaction in general. Steel and PDMS were tested with oils and lubricants in preliminary test series in order to find fluids in a suitable contact angle range. Pretests showed that oils and oily emulsions with contact angles much below 20° would lead to omnidirectional fluid movement, while contact angles above 90° would hinder the fluid from penetrating the structure and/or lead to no fluid movement at all. Therefore, the final measurements presented in this work were conducted with Sonax P809 cutting oil (Sonax, Germany) on the steel prototype. PDMS was tested with the multipurpose oil WD-40 (WD-40 Company, USA). PMMA and PI foils, on the other hand, were pretested primarily with water-based solutions, like soapy water or alcohol. Pretests again showed similar fluid behaviour for fluids with contact angles too high or too low. The ideal fluid for the PMMA was found to be 1% of soapy solution (DAWN liquid dish soap, Procter & Gamble, USA), while a 0.0075% soapy solution was chosen for the PI foil prototype. All fluids were coloured with dyes in order to improve contrast for video analysis. Water-based solutions were coloured with 0.5% of Ponceau red (Sigma Aldrich, Germany), while oil-based fluids were coloured with 0.5% of Sudan black (Sigma Aldrich, Germany).

Static contact angles were measured on a custom-made contact angle measurement set-up. The contact angles of the above-mentioned test fluids were measured on an unstructured and cleaned piece of their corresponding material, resulting in the contact angles given in table 1. The fluid volume was 3 µl for each droplet; six droplets of each fluid were measured on each corresponding material.

**Table 1.** Static contact angles for the different fluids on their corresponding test material. Static contact angle measurements were performed and the tangent half-angle (*θ*/2) method was used to calculate the contact angle. Droplet volume was 3 µl.

material/liquid	*n*	contact angle (s.d.)
PDMS/WD-40	6	23°(±2°)
steel/Sonax P809	6	24°(±1°)
PMMA/DAWN 1%	6	31°(±2°)
Pl foil/DAWN 0.0075%	6	33°(±2°)

Analysis of fluid behaviour for the PI prototype was done by applying a stretched droplet of the soapy water solution across the structured array (figure 5). The fluid movement through the structure then was filmed in a custom video booth, using a Nikon D5300 (Nikon Corp., Japan) with macro lens (AF-S Nikkor 1 : 2.8, Nikon Corp., Japan).

**Figure 4.**
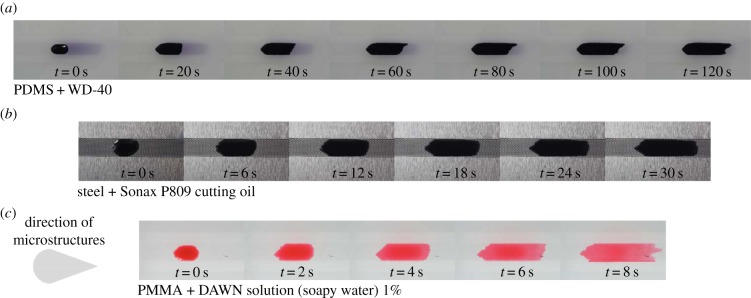
Example image rows, showing the fluid movement of the different test fluids on their corresponding artificial bug channel. (*a*) WD-40 on PDMS structure, increment between two images is 20 s. (*b*) Sonax P809 cutting oil on steel, increment between two images being 6 s. (*c*) 1% soapy water solution on PMMA, increment between images being 2 s. The direction for the microstructures within all channels is depicted in the bottom left-hand corner. Structured channel length in all cases is 5 cm.

For the video analysis of fluid movement in the upscaled prototypes, 7 µl droplets of each fluid were used for the corresponding prototype. Droplets were applied onto the channel structure using a pipette. The fluid movement was captured using the above-stated method. Each structure was tested three times with a capture frame rate of 50 fps. The video was started as soon as the fluid drop hit the structured surface and was terminated if the fluid either stopped moving on its own, or reached the end of the artificial channel. The recorded movies then were transformed into series of frame stacks, using the freeware Avidemux. Afterwards, the frame stacks were processed with a custom-made algorithm that traced the outline of the liquid front. Typical examples for the resulting frame series are shown in figure 4, where the increment between two pictures was set according to the movement speed of the different fluids on the different channel prototypes. An example video of the fluid movement for oil on structured steel is shown in electronic supplementary material, video SV1.

## 3. Results

The SEM images shown in figure 1 give a comprehensive overview of how the orientated microstructures look in the different species. The left-hand side column shows a big portion of the given ESES, composed of the ostiole (opening of the inner orifice of the gland), the peritreme (guidance structure or channel for the secreted scent fluid) as well as the evaporatorium (areas with increased surface, realized in most cases by mushroom-like microstructures). One can see that all three species, *P. prasina* (figure 1*a,b*), *R. nebulosa* (figure 1*c,d*) and *T. bicolor* (figure 1*e,f*), exhibit a full ESES with slightly varying morphological features, whereas the main focus should be placed on the microstructure that can be found in the respective peritreme.

Common for all microstructures in figure 1—besides the fact that the overall look is similar—are two main aspects: (a) the pointed tips of a group of microstructures are always orientated towards a common direction; in all cases, this direction is away from the ostiole and towards the evaporatorium; and (b) the structures are always found between the places of secretion and evaporation—a good indicator that they are involved in the liquid propagation. Based on the dimensions, which were obtained from several SEM images, abstracted three-dimensional models of the microstructure were created and used for the creation of artificial replicas, using the above-stated methods and resulting in the different prototypes from various materials and in different scales.

**Figure 5.**
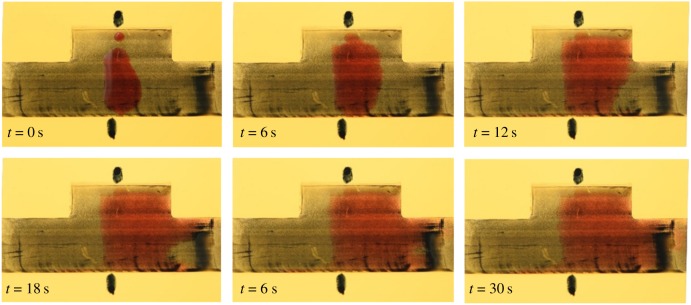
Image sequence of a PI prototype channel. A small amount of dyed soapy water (approx. 7 µl, 0.0075% soap) was applied across the structured channel array. After penetrating into the structure, unidirectional fluid movement can be seen towards the right-hand side, while it stops on the left-hand side. The pointed tip of the microstructures was towards the right-hand side in this case. The bottom edge of the structured array is 2.5 cm in length.

First prototype replicas of the bug ESES were achieved by laser structuring of PI foils, yielding microstructures with dimensions comparable to those found on the bug. The measurements from the SEM images (i.e. figure 2) yielded dimensions of length and width of 25.4 ± 4.9 µm (min. value = 8.5 µm, max. value = 41.1 µm) and 13.4 ± 3.2 µm (min. value = 5.7 µm, max. value = 25 µm), respectively. This results in an average length to width aspect ratio of 1.9 : 1, which is quite similar to the structures in the biological model. The average height of the structures can be calculated to be around 25 µm, using the known length of the structures, the angle of incidence (25.4 µm and 45°) and simple triangle calculation. This results in a height to length ratio of approximately 1 : 1, which therefore is way off the natural antetype. Testing of these samples with dyed soapy water showed pronounced unidirectional flow towards one direction, as can be seen from the image series in figure 5.

Only a small portion of the fluid is spread into the non-desired direction when reaching the bottom edge of the structured array. This is visible in the electronic supplementary material, video SV2, which shows the whole video from which figure 5 was created.

Upscaled prototypes were first measured with regard to their outcome dimensions using the Alicona images shown in figure 3. The results for all prototypes are summarized in table 2.

**Table 2.** Dimensions of microstructures, measured from the Alicona images shown in figure 3. For each replica, 10 individual structures were measured (*n* = 10); mean and standard deviation (s.d.) were calculated. All shown values are in micrometres.

	length	width	height
steel positive			
mean	342.2	150.6	77.2
s.d.	9.5	5.2	3.5
steel negative			
mean	382.6	189.1	81.4
s.d.	5.1	3.5	4.2
PMMA			
mean	339.4	168.7	68.0
s.d.	8.1	3.3	3.7
PDMS			
mean	352.0	151.0	83.4
s.d.	7.5	3.5	3.2

The values given by table 2 show that overall the dimensions of the artificial microstructures were replicated nicely, when compared with the design values given in figure 3*a*. The steel negative shows slightly exceeding values for length and width, but this is rather an effect of the energy distribution of the laser, forming a Gaussian distribution pattern, which results in rounded edges, rather than sharp ones. Hence, it was difficult to determine exactly the widest points on the falling slopes, because the PDMS copy (which shows a positive of the steel negative) does not show different values when compared with the original design. The slight shrinkage in length, while at the same time exceeding values for width, measured for the structures in PMMA are most probably explainable by the hot-embossing process and the fact that the steel negative did expand and relax in equal amounts during heating and cooling. Overall, though, the plates exhibit an aspect ratio of 2.15 (±0.14) : 1 (length : width) and 4.56 (±0.29) : 1 (length : height) (*n* = 10), which is sufficiently close to the design.

Fluid tests on these prototypes were done according to §2. Electronic supplementary material, figure S2, shows example plots of measurements that were accepted for analysis with the Matlab algorithms. Shown is the displacement of the liquid front versus the time, for the forward and backward direction, respectively. The fluids in these cases behaved like anticipated (similar to the sequences shown in figure 4), meaning that a droplet was placed carefully enough into the centre of the channels' structured part, no edge-effect occurred and a pronounced unidirectional fluid movement was observable: while the forward direction in all cases showed a pronounced slope (in most cases stronger in the beginning of the experiment and less pronounced towards the end), the curves representing the backward direction show little to no displacement of the fluid.

Besides the obvious similarities of the fluid spread between the three shown examples, individual differences are evident, the biggest one being the time scale: whereas the oil-based fluids (WD-40 and Sonax P809) show fluid movement occurring in the range of minutes, the soapy water (DAWN solution) is transported within seconds.

Something that also becomes apparent from the shown examples is the differences that occur in the stopping direction. Electronic supplementary material, figure S2A (WD-40 tested on steel), for example, exhibits stronger fluctuations for the liquid spread in the backward direction in the beginning of the experiment. Fluctuations in this case mean actual movement of the liquid front away from and towards the centre of the initially applied droplet. The longer the experiment goes on, the more steady the graph becomes, meaning that fluid movement came to a complete halt. Different behaviour is observed in the case in electronic supplementary material, figure S2B. Here one sees a relatively steady initial phase of the experiment (again, meaning no fluid displacement), with regard to the backward direction. After approximately 20 s though, fluid displacement in the backward direction starts to increase, meaning that the fluid was not stopped at that point anymore. By contrast, electronic supplementary material, figure S2C, shows relatively steady fluid flow across the whole experiment.

**Figure 6.**
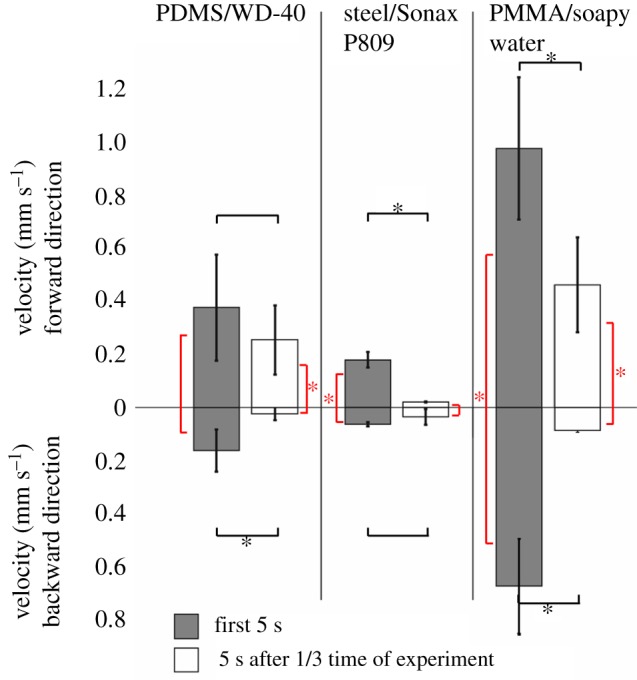
Pooled results for the liquid movement measurements on all three tested prototypes (PDMS, steel, PMMA). Depicted is the averaged velocity of the liquid front in forward (desired) and backward (undesired) directions. Dark grey bars show the averaged velocity for the first 5 s of the experiment, while white bars show the averaged velocity for 5 s after one-third of the overall experiment has passed. Student's *t*-test was used to test for significant differences (*p* = 0.05); asterisks mark significant differences; no stars means *p* > 0.05. The number of evaluated experiments for all shown test regimes was *n* = 3.

Over several different trials, all of the replica channels showed slightly different behaviour for the backward direction, sometimes resulting in steady stopping, sometimes in fluctuations in the beginning and sometimes towards the end. Overall, the behaviour nevertheless was constantly the desired one: fluid displacement in the forward direction and halting in the backward. To compare the displacement velocities in both directions, as well as the different tested systems against each other, pooled results are shown in figure 6.

Figure 6 gives a comparative overview of the fluid behaviour that was observable over the course of three evaluable experiments. The top half of the figure gives the resulting mean liquid velocities for the forward and the lower half the mean velocities for the backward direction with regard to the centre of an initially applied droplet. As the experiments showed a trend for different fluid behaviour at the beginning phases of the experiments (electronic supplementary material, S2 and figure S2), it was decided to also compare the mean velocities between the first five seconds of a measurement and the mean velocities (again of five seconds) after one-third of the experiment had passed. The individual pairs were tested against each other for significant differences via the *t*-test.

Figure 6 validates the previously assumed trends:

(1) Liquid velocities in the forward direction are always higher than in the backward direction. This behaviour is not significant for the test of the PDMS prototype with WD-40 though.
(2) Despite different time scales for the individual tested fluid/surface combinations, in general the liquid spread is faster in the beginning of an experiment when compared with the velocities after one-third of the experiment has passed. This behaviour is significant for forward and backward directions for the PMMA prototype tested with soapy water, forward direction for the steel prototype and backward direction for the PDMS prototype. Interestingly (even though not significant), the steel prototype tested with Sonax P809 showed higher velocities for the backward direction after one-third of the experiment time, than for the forward direction. This confirms what has been exemplarily shown in electronic supplementary material, figure S2B, and indicates that this trend was conserved throughout the different measurements of this prototype.

Additionally, figure 6 shows two more trends that have not been addressed before:

— Water-based test liquids seem to be transported faster through the tested channel geometry than oil-based fluids, despite exhibiting comparable contact angles on the respective channel material. This indicates that viscous forces play a role with regard to the attainable velocities.
— Water- and oil-based fluids differ in the ability to halt the fluid in the backward direction, i.e. they differ in their ability for unidirectional transport. Comparing the fluid velocities, it becomes apparent that with velocities of approximately 0.7 mm s^−1^ (soapy water) versus 0.1–0.17 mm s^−1^ (WD-40, Sonax P809) in the backward direction, the water-based solution is transported faster in the backward direction than both oils in the forward direction (despite still having a significantly faster transport in the forward direction). This holds especially true for the beginning phase of the experiments.

## 4. Discussion

### 4.1. Fluid flow on polyimide foil samples

As shown in the Results, all fabricated prototypes exhibited passive and unidirectional fluid flow, inspired by the ESES of bugs. First, simple tests of pure functionality were done with laser-structured PI foils in an attempt to copy the natural antetype as close as possible. Despite the overall positive outcome of these preliminary tests, some of the created PI samples showed slow and/or unsteady fluid flow. Additionally, as a result of the mode of production, individual structure size and density were hard to control. For this reason, upscaled and more simplified versions of the ESES microstructure were designed for laser ablation and casting. Wider spacing and omitting the undercut allowed for faster and easier creation of steel and polymer prototypes as well as more controlled testing conditions.

### 4.2. Fluid flow on upscaled samples (steel, poly(methyl methacrylate), poly(dimethylsiloxane))

The results observed on upscaled prototypes show considerable fluid-spreading velocities that were achieved on all prototypes with different fluids in one direction, while halting the fluid in the opposite. A deciding factor for whether a prototype will work with a given fluid or not was found to be the contact angle. Pretests showed that the geometry would only show pronounced, unidirectional fluid movement if the contact angles were between 20° and 35°, a requirement that was fulfilled with all tested material–liquid combinations. As shown in figure 4, fluid will always flow preferably in the direction of the pointed microstructure tips and will form a stopped, V-like shaped front in direction of the microstructures' bases.

Tracking the moving liquid front in both directions (forward and backward with regard to the centre of the initial droplet centre) and comparison of measurements in terms of fluid front displacement versus time is a perfect way to follow the dynamics of the spread (as shown in electronic supplementary material, figure S2). These findings justified not only the comparison of the total mean velocities of liquid spread, but also the division of the experiments into two stages: (1) the first 5 s (usually showing the highest spreading velocities) and (2) the first 5 s after one-third of the complete experiment.

The comprehensive compilation of results given in figure 6 shows mostly what was expected: liquid is faster transported in the forward when compared with the backward direction, and velocities are higher in the beginning of the experiment. Furthermore, a clear connection between the reachable top velocities of transport and the used fluid can be derived. Oily liquids (like WD-40 and Sonax P809) are transported slower than water-based liquids (DAWN solution). On the other hand, it seems as if the halting ability of the structure is better for oily liquids. The first trend most probably is explainable by the difference in viscosity and surface tension, because the contact angle range as well as the geometry and dimensions of the microstructures did not differ between the three tested channel prototypes.

### 4.3. Influence of viscosity and surface tension on observed fluid flow

The influence of viscosity and surface tension can be seen when looking at the analytical description of the dynamics of capillary liquid spreading in rectangular open channels as given by Berthier *et al*. [28]. In an open rectangular channel with width *w,* height *h*, the velocity of a liquid with viscosity *η* surface tension *γ* and penetration length *l* is given as4.1
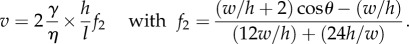


With identical geometry, i.e. with constant geometry factor *f*_2_ and the same height and length, the liquid velocity is solely dependent on the ratio of the surface tension to the viscosity. This would explain why a water-based soapy water solution (DAWN solution), with kinematic viscosity approximately 0.89 mm^2^ s^−1^ (at 25°C, source: www.viscopedia.com, 23 July 2017) and surface tension of approximately 30 mN m^−1^, would reach about three times higher velocities than, for example, WD-40 with a kinematic viscosity of 2.79–2.96 mm^2^ s^−1^ (source: WD-40 datasheet) and similar surface tension of around 31 mN m^−1^ [29]. Of course, this does not explain the observed directionality, and obviously, in reality, the liquid spread in a microstructured channel depends on more variables.

### 4.4. Flow around structures: an equilibrium approximation

To explain the basic mechanism behind the shown transport (in both PI foils as well as upscaled replicas), one needs to follow the approach of the Young–Laplace equation [30] for the pressure at a curved liquid surface (liquid meniscus):4.2
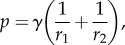
with *p* being the pressure, *γ* the surface tension, and *r*_1_ and *r*_2_ the principal radii of curvature (the maximum and minimum values, respectively, of the radius of the osculating circle; the directions of the normal plane where the radius takes its minimum and maximum are always perpendicular).

Here, we assume equilibrium and that the overall liquid pressure around a single unit cell (e.g. a single microstructure) is zero. This would be the case if the liquid were connected to an infinite liquid reservoir. In equilibrium, the liquid around a microstructure will form a so-called constant zero curvature surface if it fulfils4.3
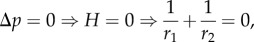
with *H* being the local curvature. A comprehensive deduction of the physics behind the observed fluid transport can, furthermore, be found in electronic supplementary material, S1.

**Figure 7.**
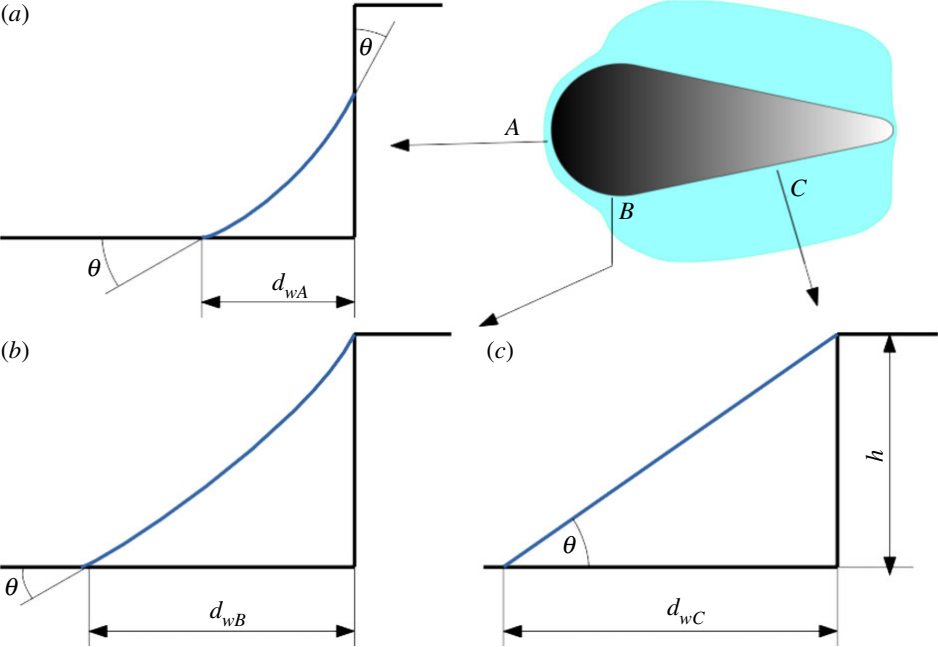
Distance of the waterfront calculated with regard to the main curvature of the microstructure at different zones (*a–c*). *θ* is the contact angle, *d_w_* is the calculated distance with regard to the corresponding zone and *h* is the height of the structure.

The walls of each microstructure meet the bottom of the channel in a perpendicular way, hence for contact angles below 45° a capillary around each microstructure is formed. Assuming that the pressure around each structure is zero and the surface tension does not change, the surface of a liquid slug in the capillary around a single microstructure is determined by the two main radii of curvature. Convex parts of the microstructure (tip and end), therefore, ‘force’ the liquid into expressing a concave radius of curvature in order to have pressure zero, hence holding the liquid front at close distance from the microstructure (figure 7*a*), while straight parts of the microstructure (figure 7*c*) allow for straight curvature of the liquid and, therefore, maximum distance of the liquid front from the microstructure. Consequently, at transitioning zones of the structure (figure 7*b*) there is a transitioning in the liquid front distance from close to far and vice versa.

**Figure 8.**
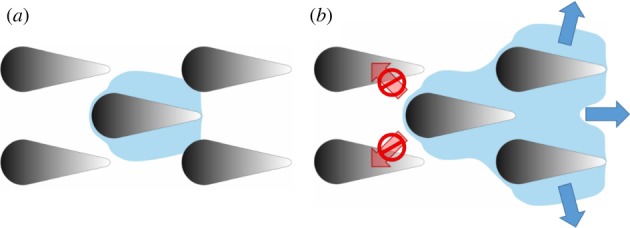
Schematic of the liquid slug around a single unit, reaching the next iteration of microstructures in the forward direction. (*a*) A single unit cell surrounded by fluid as described in figure 7. In the direction of the microstructures tip, fluid can reach the next iteration of microstructures, but not in the direction of the base. (*b*) Newly reached structures are surrounded by fluid, forming a new fluid front. Fluid can jump from iteration to iteration in the forward direction but not in backward direction, thus forming the observed V-shaped stopping front.

Following the above approach, it becomes obvious why our produced structures transported fluid unidirectionally. PI foil prototypes as well as upscaled replicas keep the liquid front at short distance at both curved ends of each individual microstructure, while the liquid front is able to reach further out at the straight part in between. Hence, the liquid front is able to reach the next iteration of microstructures in the forward direction (straight part faces a curved part), but not in backward direction (curved part faces a curved part), as also depicted in figure 8.

As long as there is fluid coming from a reservoir, this process of fluid surrounding an individual microstructure in the above-depicted way, reaching towards the next iteration of microstructures in the forward direction and so on, is repeated until the microstructured channel is filled or the reservoir is depleted. This also explains the always-observed V-shape in the stop direction.

For reasons of efficiency, it appears desirable for the bug not to waste much of the defensive fluid for filling the liquid-guiding structure. Thus, we estimate the amount of liquid that is at least necessary for filling (or that stays as a minimal film in) the peritreme, while not reaching the evaporatorium.

For a continuous film in the channels in between the microstructures we assume the surface of the liquid to form a circular (cylindrical) meniscus in cross section, intersecting the vertical structure wall at the contact angle *θ*. If two microstructures are separated by a distance *l*, the radius *r* of this meniscus is4.4
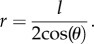


Simple geometric considerations yield a minimal height *h*_min_ of the liquid at the walls of4.5
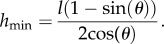


This height at the wall is reached when the liquid meniscus barely touches the bottom of the channel in the middle of the channel, i.e. at *l/*2.

Now, given the average measures of the geometric parameters described above, we can estimate the minimal height at the walls as follows: for a contact angle of 40° and a channel width of 5 µm, the minimal height *h*_min_ ≈ 1 µm at the wall. Thus, the average height in the channel for forming the minimal liquid can be found by integrating, yielding approximately *h*_av_ ≈ 0.35 µm. Thus, the minimal volume follows to be4.6
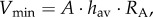
with *A* denoting the total area and *R_A_* denoting the area ratio of channels versus structures. If now the area of interest is 10 000 µm^2^ (corresponding to 100 × 100 µm^2^) and the area of the channels is 60% of the total area, one obtains a volume of approximately 2000 µm^3^, which equals 2 × 10^−6^ µl.

## 5. Conclusion

The ESES of selected European true bugs indeed seems to be a promising source for ideas to come up with novel solutions for passive and directed fluid transport on technical surfaces. We showed in different scales of dimension, as well as with different fluid–material combinations that the underlying principles of equipotential-driven fluid transport are freely applicable, as long as the boundary conditions are fitting (e.g. contact angle < 45°, correct spacing and shape of microstructures). Having understood the operating mode of ESES and artificial structures, future work will allow for improved and modified structures, because in nature, most specializations usually serve a multitude of functions (in case of the ESES, the microstructure probably also serves as hindrance for parasites and pathogens) and, therefore, do not represent necessarily the optimum. The findings of this work will aid all fields in which passive and unidirectional fluid movement is of concern. Especially microfluidics as well as lubrication and wear resistance applications seem to be a suitable target.

## Supplementary Material

S1

## Supplementary Material

S2

## Data accessibility

All data supporting this study are provided as electronic supplementary material accompanying this contribution.
