# Supplementary material for: The external scent efferent system of selected European true bugs (Heteroptera): a biomimetic inspiration for passive, unidirectional fluid transport
Source: J R Soc Interface. 2018 Mar 28;15(140):20170975. doi: 10.1098/rsif.2017.0975 (PMC5908534; doi:10.1098/rsif.2017.0975)
Supplement: S1 [file rsif20170975supp1.pdf]

Flow around structures: An equilibrium approximation

The structures that protrude from the surface can be described as general cylinders which is defined as a surface consisting of all the points on all the lines which are parallel to a given line and which pass through a fixed plane curve in a plane not parallel to the given line. In our case the droplet-shaped footprint is the fixed planar curve and the parallel lines defining the cylinder are perpendicular to the surface of this footprint. These general cylinders are truncated at a given height above the surface.

If now liquid is brought in contact with such a general cylinder and the base surface respectively, the liquid surface will form in such a way that the energy of the system is minimized. The surface of the liquid can be described by a function *f(x,y)* over the surface plane, where x and y are the planar coordinates. For a general function *f(x,y)* the local curvature *H* is defined as

$$H=-\frac{1}{2}\cdot\nabla\cdot\left(\frac{\nabla F}{\|\nabla F\|_2}\right)=\frac{\left[1+\left(\frac{\partial f}{\partial x}\right)^2\right]\frac{\partial^2 f}{\partial y^2}-2\cdot\frac{\partial f}{\partial x}\cdot\frac{\partial f}{\partial y}\cdot\frac{\partial^2 f}{\partial x\partial y}+\left[1+\left(\frac{\partial f}{\partial y}\right)^2\right]\frac{\partial^2 f}{\partial x^2}}{2\cdot\sqrt{\left[1+\left(\frac{\partial f}{\partial x}\right)^2+\left(\frac{\partial f}{\partial y}\right)^2\right]^3}} \qquad (1)$$

which is connected to the Laplace-pressure of the liquid as

$$\Delta p=\frac{\partial E}{\partial V}=\gamma\cdot\left(\frac{1}{r_1}+\frac{1}{r_2}\right)=2\cdot\gamma\cdot H \qquad (2)$$

where r_1 and r_2 are the main radii of curvature. If the liquid is connected to an infinite liquid reservoir at pressure 0, then the liquid surface will form a so called constant zero curvature surface, also called minimal surface with fulfils

$$\Delta p=0 \quad\Rightarrow\quad H=0 \quad\Rightarrow\quad \frac{1}{r_1}+\frac{1}{r_2}=0 \qquad (3)$$

If the contact angle of the liquid-solid combination is below 45°, energetically stable liquid surfaces can be found, that fulfil the above condition. In general it is hard to find minimal surfaces based on arbitrary boundary conditions. In our case the boundary conditions is the contact angle at which the liquid interacts with the solid surface.

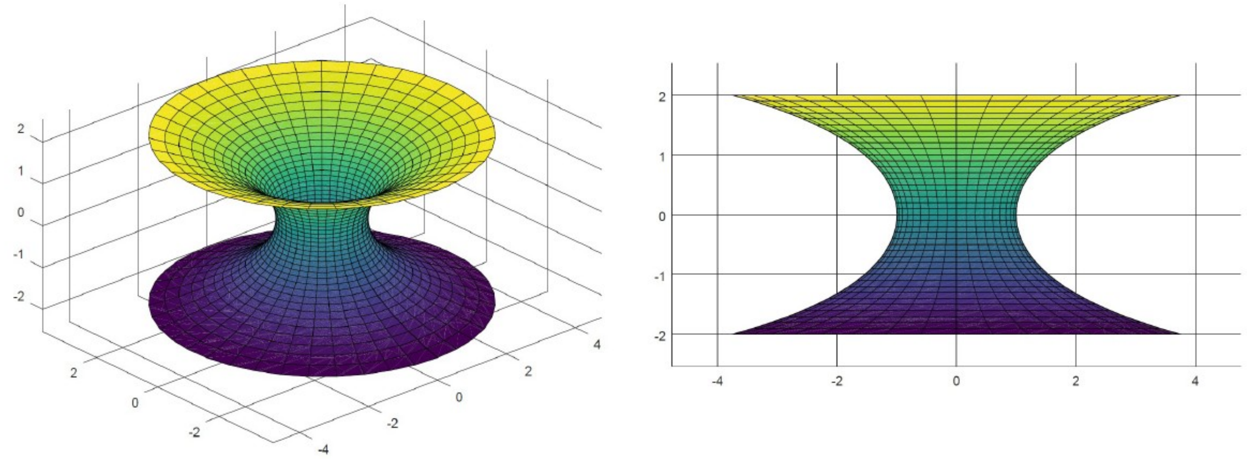

Figure S1: Part of a catenoid with center axis in y-direction at x=0 and z=0 with radius *a*=1 at bird’s eye view (left) and side view (right).

In the case of a right circular cylinder (curve in the plane is a circle), a simple solution for the minimal surface problem given the contact angle can be found to be a catenoid. A catenoid is a surface arising by rotating a catenary curve about an axis. The catenoid may be defined by the following parametric equations:

$$x = a \cdot \cosh\frac{v}{a} \cdot \cos u \quad y = a \cdot \cosh\frac{v}{a} \cdot \sin u \quad z = v \tag{4}$$

here $u \in [-\pi, \pi[\,, v \in \mathbb{R}$ and a is a non-zero real constant which is the radius of curvature of the catenary curve at the vertex as well as the radius for the rotation of the vertex around the z-axis.
In figure S1 a catenoid can be seen in two views with a=1. Clearly the catenoid has a constant mean curvature of zero.
Now for the right circular cylinder of finite height h we have to distinguish two cases. The first one is depicted in Figure S2.

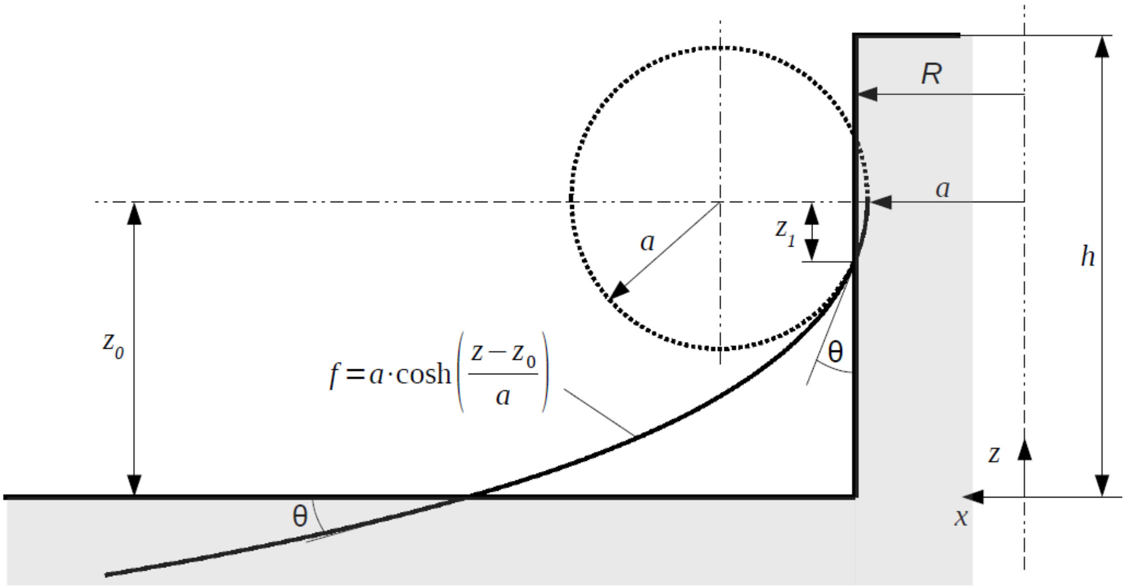

Figure S2: Case one for a catenary curve *f(z)* forming a catenoid when rotating around the z-axis. A right circular cylinder with radius *R* as well as the base surface are intersected at an angle θ, the contact angle.

Here the catenoid with vertex radius of curvature a intersects with the cylinder as well as with the surface, i.e. the xy-plane. Now the radius of the cylinder R and the contact angle θ are given and the parameter (radius) a of the catenary curve and the z-position of the vertex z_0 as well as the distance to the intersection z_1 have to be determined. For this we need the fact that

$$\frac{df}{dz} = \sinh\left(\frac{z - z_0}{a}\right) \tag{5}$$

Applying this at the intersection of catenoid and cylinder, i.e. at a distance z_1 from the vertex yields

$$z_1 : \quad \sinh\left(\frac{y_1}{a}\right) = \tan\theta \quad \Rightarrow \quad z_1 = a \cdot \operatorname{arcsinh}(\tan\theta) \tag{6}$$

At the intersection of catenoid and cylinder the function f equals the cylinder radius R, i.e. $R = a \cdot \cosh(z_1 / a)$ thus by inserting z_1 from Eq. 6 we obtain the analytical solution

$$a = \frac{R}{\cosh\left[\operatorname{arcsinh}\left(\tan\theta\right)\right]} \tag{7}$$

Now the position z_0 of the vertex can be found

$$\sinh\left(\frac{z_0}{a}\right) = \cot(\theta) \quad \Rightarrow \quad y_0 = a \cdot \operatorname{arcsinh}\left[\cot(\theta)\right] \tag{8}$$

These relationships derived so far hold true if the height of the cylinder is larger than the z-position of the vertex, i.e. if $h > z_0$. If this is true, then the above derived iteration in Eq. 7 converges and real solutions for a and z_0 can be found. A solution for the catenoid forming the liquid surface around a cylinder of radius 1.1 is shown in Figure S3.

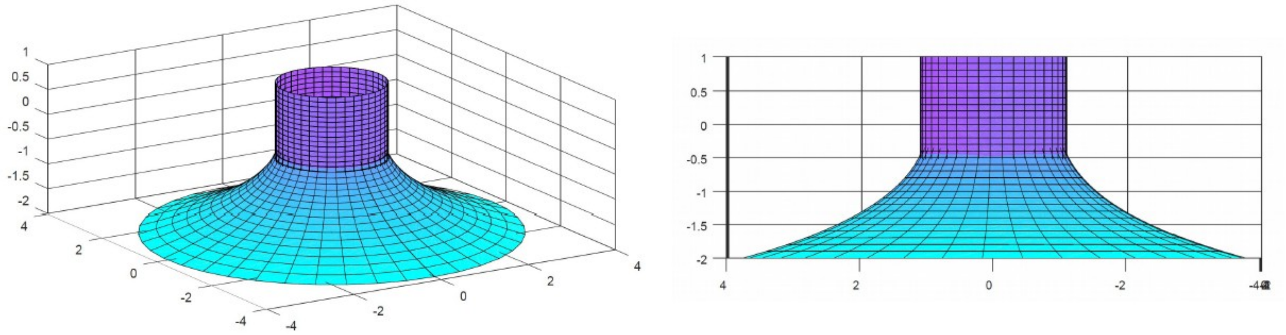

Figure S3: Liquid surface (blue) formed around a cylinder (violet) of radius 1.1 in bird’s eye view (left) and side view (right).

If the radius of the cylinder R is too large or the height h is too low, another solution has to be found. The principle is depicted in Figure S4.

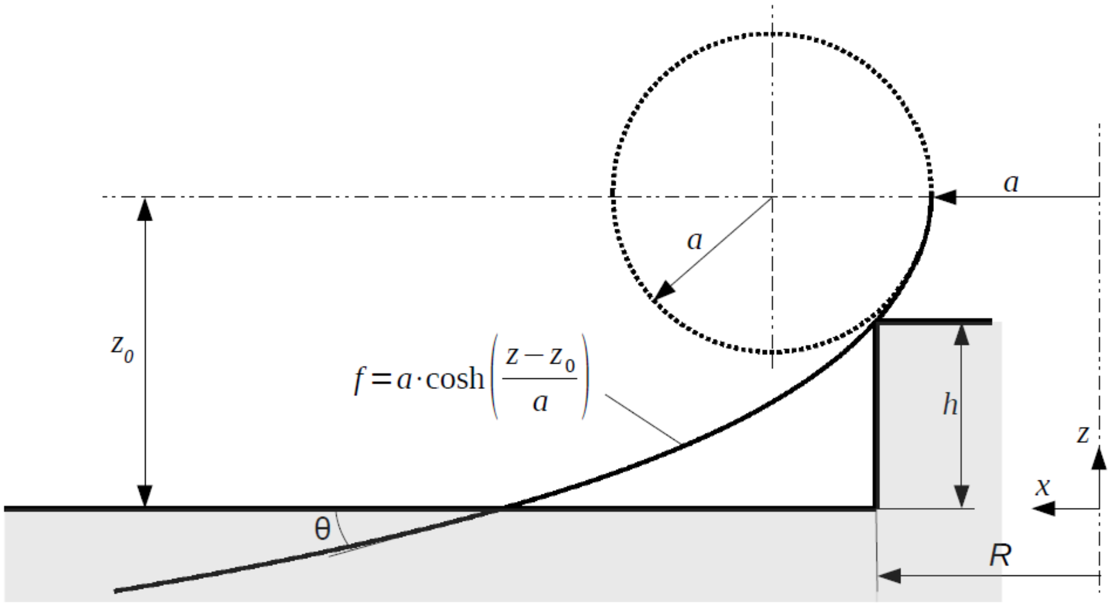

Figure S4: Case two for a catenary curve *f(z)* forming a catenoid when rotating around the z-axis. The base surface is intersected at the contact angle θ. The upper edge of the cylinder forms a singularity, i.e. it is part of the catenary curve.

Here the edge of the cylinder, i.e. the position $(x,z)=(R,h)$ is located on the catenary curve, thus we find

$$R = a \cdot \cosh\left(\frac{y_0 - h}{a}\right) \tag{9}$$

In the current case Eq. 8 is also valid. Combining Eq. 8 and Eq. 9 we obtained

$$a = R \cdot \cosh\left\{\frac{a \cdot \sinh[\cot(\theta)] - h}{a}\right\}^{-1} \tag{10}$$

This implicit equation for a given R and θ can simply be solved by iteration with a start value for a to be set to R. Typically we reach convergence to a precision of <1% after 3 to 10 iterations for 25°<θ<44° and R>0.
The calculation of the z-position of the vertex can be done according to Eq. 8.

This second case where the liquid meniscus hits the upper edge of the structure at $z=h$ contains the special case that $R \rightarrow \infty$, i.e. that the structure has a straight wall. However, this case is rather simple to treat as the solution is a plane that intersects the bottom at the contact angle θ and that hits the upper edge of the structure. This corresponds to case C in Figure 8 of the manuscript.

Now in order to find the average mean zero curvature surface the liquid forms around a general cylinder with a more or less arbitrary footprint, we used the following approximation: If the footprint (in our case the droplet-shape) exhibits no absolutely abrupt changes in the curvature, i.e. if it is at least twice continuously differentiable, the surface is locally approximated by a cylinder with the radius of the local radius of curvature of the footprint. For this cylinder the intersection of the corresponding catenoid with the xy-plane is calculated. The intersection of the catenary curve in the plane perpendicular to the tangent plane on the general cylinder is found. This point is marked. The procedure is repeated for a finite set of points along the footprint of the structure. Finally the found points are interpolated. Thus we approximate the structure locally by cylinders and smoother the catenoid-surfaces in order to find a good approximation for the minimal surface surrounding the general cylinder.
This can be done for rather general footprints as long as the curvature changes moderately along the outline. Either the shape is given locally by analytic functions (biarcs, splines, hyperbolas, …) from which the local curvature can be directly derived, or the footprint is given by a set of xy-coordinates of supporting-points. From these the local curvatures and the tangent planes can be estimated numerically.

This was done for the droplet-shaped structures of the bark bugs as shown in Figure 7 and 8 of the main manuscript. There the intersection point of the catenoid with the xy-plane, i.e. f(z=0) is shown for the given structure, indicating the asymmetric fluid behaviour.
