# Supplementary material for: The external scent efferent system of selected European true bugs (Heteroptera): a biomimetic inspiration for passive, unidirectional fluid transport
Source: J R Soc Interface. 2018 Mar 28;15(140):20170975. doi: 10.1098/rsif.2017.0975 (PMC5908534; doi:10.1098/rsif.2017.0975)
Supplement: S2 [file rsif20170975supp2.pdf]

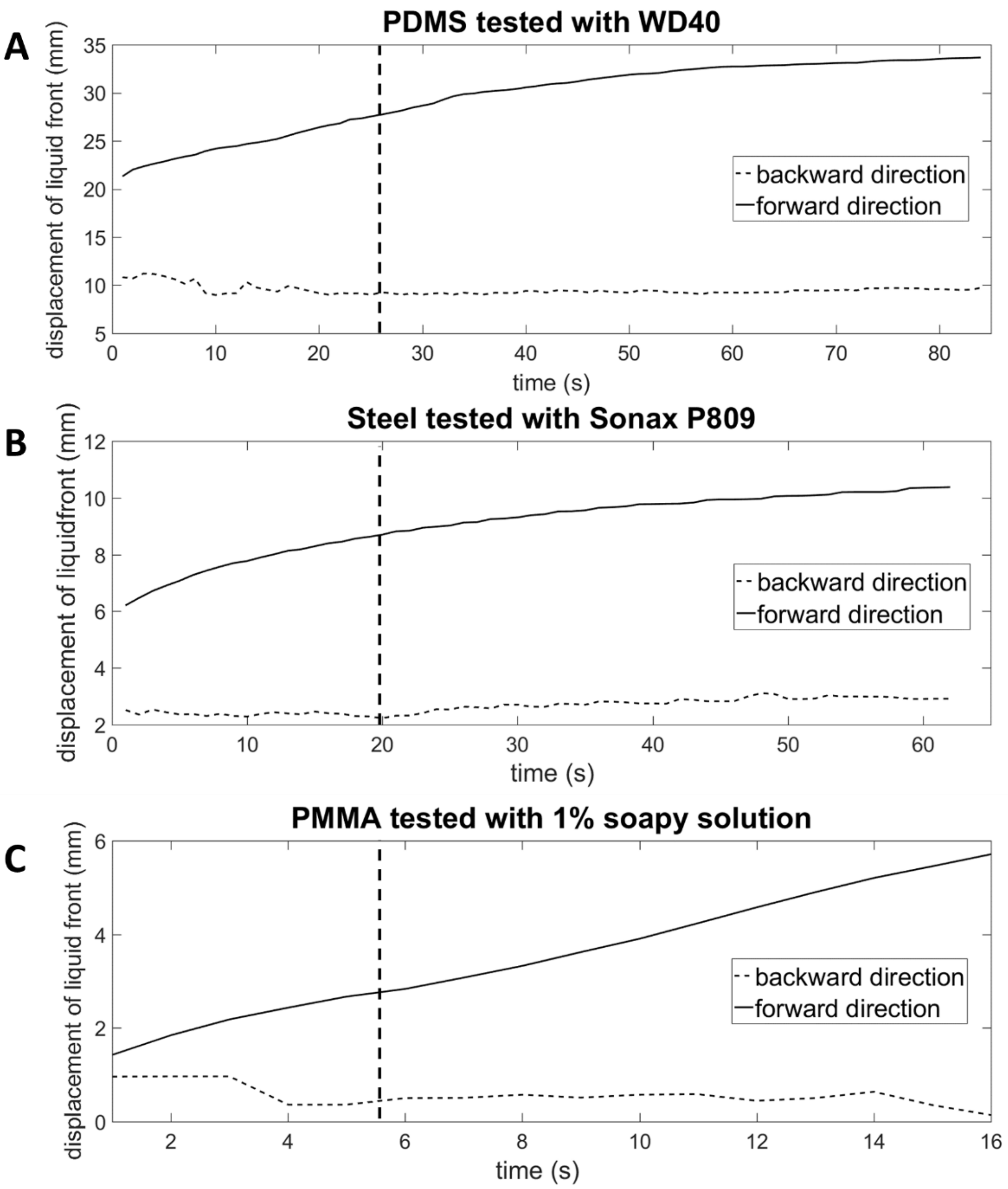

Figure S2: Comparison of typical results of fluid movement in forward and backward direction. *Shown is the displacement of the liquid front (with regard to the initial droplet center = center of wetted area) versus the time. Solid lines indicate the fluid front in forward direction, dashed lines indicate backwards.* ***A*** *Example result for the liquid transport of WD-40 on micro-structured PDMS.* ***B*** *Example result for the liquid transport of Sonax cutting oil on micro-structured steel.* ***C*** *Example result for the liquid transport of soapy water solution (1%) on micro-structured PMMA. Perpendicular dashed lines mark the point of time when 1/3 of the overall experimental time had passed.*
